# Human Quadrupeds, Primate Quadrupedalism, and Uner Tan Syndrome

**DOI:** 10.1371/journal.pone.0101758

**Published:** 2014-07-16

**Authors:** Liza J. Shapiro, Whitney G. Cole, Jesse W. Young, David A. Raichlen, Scott R. Robinson, Karen E. Adolph

**Affiliations:** 1 Department of Anthropology, University of Texas at Austin, Austin, Texas, United States of America; 2 Department of Psychology, New York University, New York, New York, United States of America; 3 Department of Anatomy and Neurobiology, Northeast Ohio Medical University, Rootstown, Ohio, United States of America; 4 School of Anthropology, University of Arizona, Tucson, Arizona, United States of America; Scientific Institute Foundation Santa Lucia, Italy

## Abstract

Since 2005, an extensive literature documents individuals from several families afflicted with “Uner Tan Syndrome (UTS),” a condition that in its most extreme form is characterized by cerebellar hypoplasia, loss of balance and coordination, impaired cognitive abilities, and habitual quadrupedal gait on hands and feet. Some researchers have interpreted habitual use of quadrupedalism by these individuals from an evolutionary perspective, suggesting that it represents an atavistic expression of our quadrupedal primate ancestry or “devolution.” In support of this idea, individuals with “UTS” are said to use diagonal sequence quadrupedalism, a type of quadrupedal gait that distinguishes primates from most other mammals. Although the use of primate-like quadrupedal gait in humans would not be sufficient to support the conclusion of evolutionary “reversal,” no quantitative gait analyses were presented to support this claim. Using standard gait analysis of 518 quadrupedal strides from video sequences of individuals with “UTS”, we found that these humans almost exclusively used lateral sequence–not diagonal sequence–quadrupedal gaits. The quadrupedal gait of these individuals has therefore been erroneously described as primate-like, further weakening the “devolution” hypothesis. In fact, the quadrupedalism exhibited by individuals with UTS resembles that of healthy adult humans asked to walk quadrupedally in an experimental setting. We conclude that quadrupedalism in healthy adults or those with a physical disability can be explained using biomechanical principles rather than evolutionary assumptions.

## Introduction

In 2005, Uner Tan described a Turkish family with 19 siblings, five of whom (ages 14–32 years) exhibited “Uner Tan Syndrome” or “UTS”, characterized by impaired cognitive abilities, dysarthric speech, cerebellar hypoplasia, and habitual quadrupedal gait on hands and feet. Tan [1] interpreted the symptoms exhibited by these individuals as an example of “human devolution”, stating that they “may provide us with some important clues about the transition from quadrupedality to bipedality, along with the evolution of the human mind” (page 251 [1]). A key claim used in support of the “devolution” hypothesis is that the form of quadrupedalism used by individuals with UTS resembles that of nonhuman primates (hereafter referred to as ‘primates’), representing a reversal to a “primitive” state. Specifically, individuals with UTS have been described by Tan and colleagues as using diagonal sequence gait, the form of quadrupedalism that sets primates apart from almost all other mammals [2]–[8].

After the initial identification and description of Uner Tan Syndrome, Tan and colleagues published an extensive series of papers further documenting evidence of UTS in other individuals [1], [9]–[20]. A mutation in very-low-density lipoprotein receptor gene was said to be the cause of both the cerebellar hypoplasia and quadrupedal locomotion in at least some of these individuals [21], although genetic causes of UTS were reported to be heterogeneous [13], [22]–[24]. Because the presence of quadrupedalism is variable with respect to the identified genetic mutations, other researchers have argued that the use of quadrupedalism is not the direct effect of genetic mutation, but an adaptation to instability of the trunk caused by (genetically determined) cerebellar dysfunction, combined with environmental conditions such as insufficient medical care [25]–[30]; but see [22], [31]. Although a recent paper acknowledged that “the genetic associations hitherto reported for the UTS seem to have no or only minor explanatory power, if any, for the origins of human quadrupedalism”(page 89 [32]), and acknowledged the role of other factors such as socioeconomic status, researchers continue to make persistent (but unsupported) claims regarding the “primitive” and “primate-like” nature of the quadrupedalism [1], [9], [11], [13], [14], [17]–[20], [31]. In this paper, we show that the quadrupedal kinematics exhibited by humans with UTS has been erroneously described as primate-like by the authors, further weakening the “devolution” hypothesis put forth in this series of papers.

Although the diagnosis of Uner Tan syndrome was extended to individuals who lacked some of the symptoms (e.g., not all showed cerebellar atrophy or mental impairment) [10], [11], all were identified as using quadrupedal walking either habitually (if bipedalism was physically untenable), or, even when capable of bipedalism, as a preferred method of locomotion at particular speeds or in certain situations [17], [18]. Since quadrupedalism is the only physical manifestation common to all individuals diagnosed with UTS (truncal ataxia is also common to individuals with UTS, but truncal ataxia is associated with related syndromes such as disequilibrium syndrome (DES), and is therefore not exclusive to UTS [18]), the habitual or intermittent use of this form of locomotion can be viewed as the foundation of the claim by Tan and colleagues that these humans exhibit evidence of evolutionary reversal or “devolution.” Moreover, Tan and colleagues have consistently claimed that these individuals are, more specifically, using a form of quadrupedal walking characteristic of nonhuman primates (diagonal sequence quadrupedalism), presumably linking humans with UTS to our evolutionary past [1], [9], [11], [13], [14], [17]–[20], [31].

The response to these papers has focused mainly on whether the quadrupedalism is genetically determined or simply an adaptive response to the impaired ability to walk bipedally in individuals with a genetic mutation [25]–[30]. To date, there has been no response to the repeated claims that the people with UTS are using primate-like diagonal sequence quadrupedalism, despite the fact that Tan and colleagues have never presented data supporting this key element of their “devolution” hypothesis.

**Figure 1:**
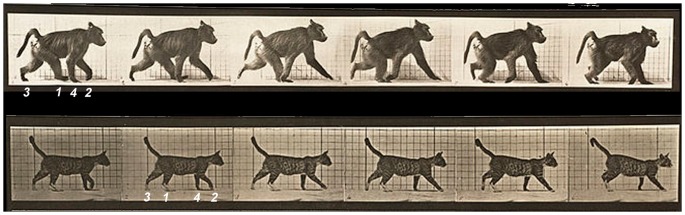
Comparison of footfall sequence in primate (baboon, above) and nonprimate (cat, below). Footfall sequence is depicted numerically, beginning with the right hind limb in each animal. The primate is walking in diagonal sequence (RH-LF-LH-RF), and the nonprimate is walking in lateral sequence (RH-RF-LH-LF), where R = right, L = left, H = hind limb, and F = forelimb. Images from [2].

The distinctiveness of primate quadrupedal gait compared to that of most other mammals is well documented [2]–[8]. Although primates exhibit some flexibility, they prefer a diagonal sequence/diagonal couplets (DSDC) walking gait [4]–[8], [33]–[37]. Most other mammals prefer a lateral sequence gait, with either diagonal or lateral couplets (LSDC or LSLC), depending on the animal’s limb proportions [7], [8], [38]. The distinction between *sequence* and *couplets* is important for understanding quadrupedal gait, but this distinction has not been made in the literature documenting Uner Tan Syndrome. Sequence refers to the order of footfalls. In a diagonal sequence walk, a hind limb touches the ground, followed by the contralateral (opposite side) forelimb. In a lateral sequence walk, a hind limb touches the ground, followed by the ipsilateral (same side) forelimb (Fig. 1). Either sequence can be produced by diagonal or lateral couplets. When couplets are diagonal, the contralateral fore- and hind limb swing and land close together in time. When couplets are lateral, the ipsilateral fore- and hind limb swing and land close together in time. Mammals also use walking gaits in which ipsilateral (pace) or contralateral (trot) forelimbs and hind limbs land simultaneously, or “singlefoot” gaits (in lateral or diagonal sequence) in which all four limbs are equally spaced in time, rather than paired in couplets [38], [39].

Hildebrand [38], [39] devised a method by which footfall sequence and interlimb timing (e.g., couplets) can be quantitatively described by calculating “limb phase”– the percentage of a hind limb’s stride duration that the touchdown of a forelimb follows that of the hind limb on the same side of the body (see Methods for details). Hildebrand’s extensive analyses of gait across mammals and other tetrapods demonstrated that during quadrupedal walking, the limb phases most commonly used by mammals correspond to lateral sequence, diagonal couplets (LSDC) and lateral sequence, lateral couplets (LSLC) gaits. Primates are distinctive for preferring diagonal sequence, diagonal couplets (DSDC) walking gaits, but there are a few other mammals whose walking gaits fall into the DSDC category (e.g., kinkajous, giant armadillos, aardvarks and some arboreal marsupials) [38], [40]–[43].

Although the use of primate-like, DSDC quadrupedal gait in humans (as opposed to the forms of quadrupedalism used by most nonprimate mammals, LSDC or LSLC) would not be sufficient to support the conclusion of evolutionary “reversal”, Tan and colleagues have not conducted any quantitative analyses of the gait sequences exhibited by these individuals. Nor have Tan and colleagues examined this quadrupedalism in the context of the numerous studies that have provided quantitative kinematic data on healthy human adults (as well as infants and children) asked to walk quadrupedally in an experimental setting [4], [44]–[48]. Therefore, to date, it has not yet been empirically determined whether the human individuals reported to have UTS use a form of quadrupedalism similar to that of nonhuman primates, or how their quadrupedal walking compares to that of healthy human participants.

The purpose of this paper is to use standard quantitative methods to test the assertion repeatedly put forth in the literature that the form of quadrupedalism used by humans with Uner Tan Syndrome is “primate-like.” We then discuss the results in the context of other quantitative studies on quadrupedalism in healthy human adults, infants and children, and address the claim made by Uner Tan and colleagues that the quadrupedalism used by these individuals represents an evolutionary “reversal” to a more primitive primate state.

## Methods

Video sequences of humans using quadrupedalism were obtained from two sources:

1. Footage (filmed at 25 frames/sec) of the siblings from the Turkish family in which UTS was first described and defined (“Family A”; [13]), was provided by Passionate Productions, portions of which were aired on the BBC2 documentary “The Family that Walks on All Fours.” From this source, 513 symmetrical walking strides were analyzed, from five individuals, one male and four females (Participants 1–5; Table 1).
2. Video sequences of humans with “UTS” walking quadrupedally from families other than the one documented in #1 were obtained from supplementary data accompanying [13] (15 frames/sec) and [28] (29.97 frames/sec). After excluding data from asymmetrical strides (see further explanation below), strides from an individual with paralysis in one leg, and strides not consistently visible in the videos, we present limited data from two individuals (Participants 6 and 7, Table 1). Participant 6 (n = 4 strides) is a twelve year old boy with truncal ataxia who walked upright but developed “facultative quadrupedal gait for fast locomotion” at the age of ten [13]. Participant 7 (n = 1 stride) is an adult male who walked on hands and feet from infancy, and who was able to walk bipedally for a few steps but with difficulty balancing [28]. Due to the small size of this additional sample, statistical analyses were restricted to the data obtained from Family A (#1 above). Supplementary videos from other publications were either not available online [11], [12], were provided by publisher in a format (.wcp) not readily viewable [9], or were of insufficient quality for reliable quantitative analysis [15], [20].

**Table 1:** Sample.

Participant	Sex	Age	Strides	Source
1	M	adult	332	Passionate Productions (Family “A”)†
2	F	adult	72	Passionate Productions (Family “A”)
3	F	adult	27	Passionate Productions (Family “A”)
4	F	adult	76	Passionate Productions (Family “A”)
5	F	adult	6	Passionate Productions (Family “A”)
6	M	12 years	4	Video accompanying [13]
7	M	adult	1	Video accompanying [28]

†Family A as described in [13].

Permission for use of the footage in #1 was granted by Passionate Productions. All video sequences used in this analysis (from the Passionate Productions footage or from supplementary videos accompanying published articles) were in the public domain and thus Human Subjects Institutional Review Board permission was not required. Sample sizes by participant are listed in Table 1.

### Quantification of footfall sequence and interlimb timing using limb phase

Limb phase was measured as the percentage of a hind limb’s stride duration that the touchdown of a forelimb follows that of the hind limb on the same side of the body. As defined, limb phases fall on a continuum between 0 and 100%, but ranges of limb phase values can be used to express more discrete gait categories. Following Cartmill et al. [7], limb phases of 0% or 100% correspond to a pace, 50% corresponds to a trot, and 25% and 75% correspond to singlefoots (in lateral or diagonal sequence, respectively). Limb phase values between 0 and 25% correspond to lateral sequence, lateral couplets (LSLC), those between 25% and 50% correspond to lateral sequence, diagonal couplets (LSDC), those between 50% and 75% correspond to diagonal sequence, diagonal couplets (DSDC), and those between 75% and 100% correspond to diagonal sequence, lateral couplets (DSLC). In contrast, Hildebrand represented named gait categories by dividing the 0–100% limb phase range into octiles in part because “a trained observer can distinguish by eye between ways of moving that differ on either scale of the graph by 10 to 15 percentage points” (page 216 [38]). Rather than base our categories on (subjective) units visible to the trained eye, we opted to use Cartmill et al.’s [7] slight modification of Hildebrand’s gait categories because they more strictly represent the coordination of the limbs. For example, in Cartmill et al’s method [7], a value of 50% represents a true trot (diagonal limbs landing simultaneously), and values between 50% and 75% represent diagonal sequence/diagonal couplets. Thus, a limb phase of 51% would indicate a slight offset from a trot, with the limbs moving in diagonal sequence. In Hildebrand’s method [38], values for DSDC are 56.25%–68.75%, and thus, a value of 51% is considered a trot rather than diagonal sequence, even though the limbs are moving in a diagonal sequence. Our data are analyzed using Cartmill et al.’s method, but a comparison of results using the two methods can be found in Table S1.

Walking gaits were defined as those in which the mean duty factor (the percentage of a stride’s duration that a limb is on the ground) of all four limbs was greater than or equal to 50% [39]. Only symmetrical walking strides were included because distinctions between the named gaits above are relevant only for symmetrical strides. Symmetry was calculated as the percentage of a limb’s stride duration that the contralateral limb reached midstance. In a perfectly “symmetrical” walk, this value would be 50% [38]. Because perfect symmetry is rare, we accepted strides for which fore and hind limb symmetry values were between 40% and 60%. Dataset S1 lists kinematic variables for all participants and strides.

To examine the relation between limb phase and speed, limb phase was plotted against mean duty factor [38]. Differences in gait frequencies were tested with a chi-squared test. Mean duty factor was positively correlated with limb phase across the sample, and within three of the five individuals (see below). Due to the fact that there was a significant interaction between mean duty factor and individual, we could not conduct an analysis of covariance to control for the effect of duty factor on limb phase. Therefore, we used ANOVA in conjunction with post hoc multiple comparisons to test for differences in limb phase among individuals, restricting the comparison to overlapping ranges of duty factor (0.65–0.80). Statistical analyses were performed using SPSS V.20 (IBM Corp., 2011).

## Results

### Family “A”

**Figure 2:**
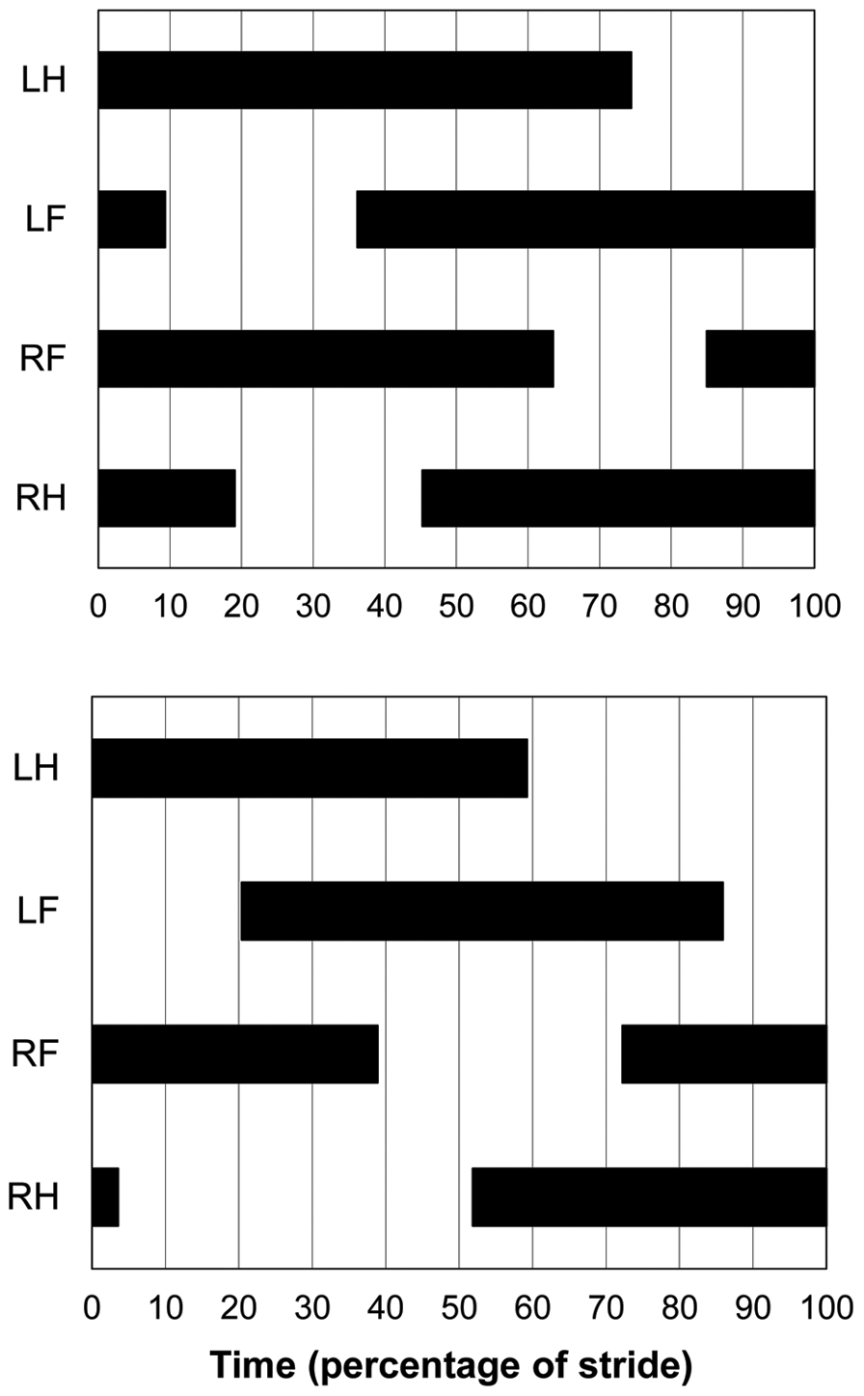
Representative footfall sequences in Participant 1 (Family “A”). Above, lateral sequence, diagonal couplets (limb phase = 0.36, mean duty factor = 0.75); below, lateral sequence, lateral couplets (limb phase = 0.20, mean duty factor = 0.61) Black bars represent the period of substrate contact for each limb (LH: left hind limb; LF: left forelimb; RF: right forelimb; RH: right hind limb).

**Figure 3:**
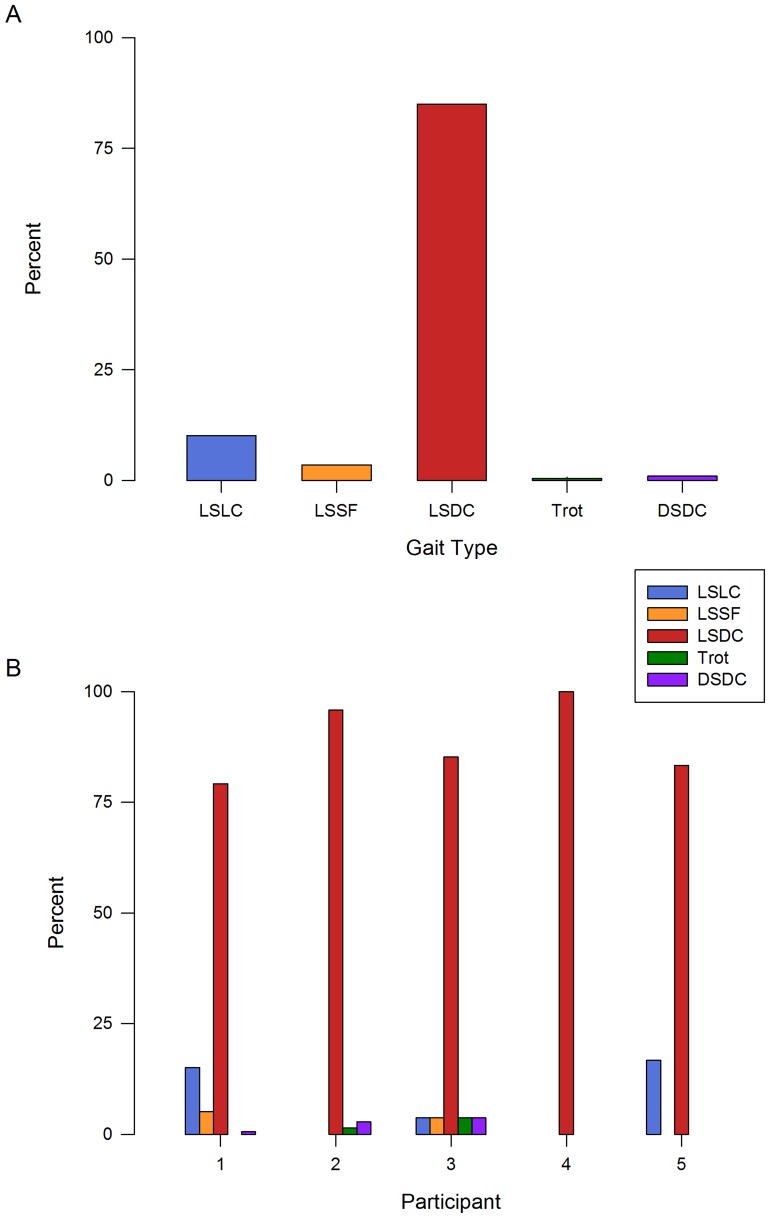
Frequencies of gait type in Family “A”. a) Total gait frequencies, and b) gait frequencies by participant. LS = Lateral sequence, DS = Diagonal sequence, LC = Lateral couplets, DC = Diagonal couplets, SF = Singlefoot.

Contrary to the published claims that the Turkish individuals with UTS used “primate-like” (DSDC) walking gaits, only 1% of the 513 strides quantified across Participants 1–5 from Family “A” were categorized as DSDC. Rather, these participants nearly exclusively (98.6% of strides) walked in lateral sequence. Among the lateral sequence strides, 85% were in diagonal couplets (LSDC), 3.5% were in singlefoot (LSSF), and 12% were in lateral couplets (LSLC) (Table 2; Figs. 2, 3). The strong preference for LSDC gaits was exhibited across the sample as a whole (Fig. 3a; *p*<0.001) and also within each individual (Fig. 3b; all *p*<0.001 except Participant 5, *p = *0.102; Table 3). Participant 4 used LSDC exclusively. Results are similar (92% of strides are LS, with LSDC the most frequent type of LS gait) if gait categories are defined using Hildebrand’s method (Table S1).

**Table 2:** Frequencies of gait type, all participants from Family “A” combined.

Gait type	Frequency	Percent
LSLC	52	10.1
LSSF	18	3.5
LSDC	436	85.0
TROT	2	0.4
DSDC	5	1.0
Total	513	100

LS = Lateral sequence, DS = Diagonal sequence, LC = Lateral couplets, DC = Diagonal couplets, SF = Singlefoot.

**Table 3:** Frequencies of gait type by participant (Family “A”).

Participant	Gait type	Frequency	Percent
1	LSLC	50	15.1
	LSSF	17	5.1
	LSDC	263	79.2
	DSDC	2	0.6
	Total	332	100.0
2	LSDC	69	95.8
	TROT	1	1.4
	DSDC	2	2.8
	Total	72	100.0
3	LSLC	1	3.7
	LSSF	1	3.7
	LSDC	23	85.2
	TROT	1	3.7
	DSDC	1	3.7
	Total	27	100.0
4	LSDC	76	100.0
5	LSLC	1	16.7
	LSDC	5	83.3
	Total	6	100.0

LS = Lateral sequence, DS = Diagonal sequence, LC = Lateral couplets, DC = Diagonal couplets, SF = Singlefoot.

**Figure 4:**
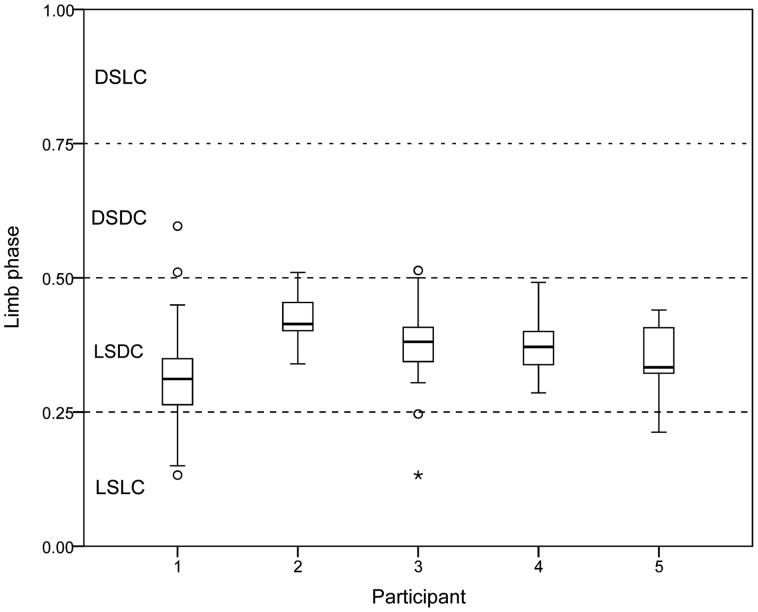
Box and whiskers plots of limb phases by participant (Family “A”). Lines represent the median, boxes represent the interquartile range, whiskers are 1.5x the interquartile range, circles are outliers and asterisks extreme outliers. Abbreviations as in Fig. 3.

**Figure 5:**
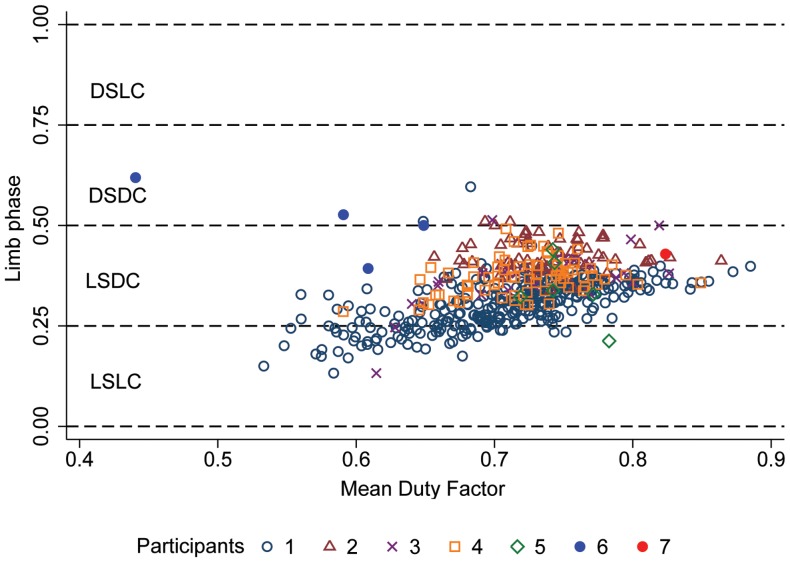
Limb phase vs. mean duty factor by participant. Participants 1–5 are from Family “A”; additional cases are represented by Participants 6 and 7 (see Table 1). Abbreviations as in Fig. 3.

Although each participant used LSDC more frequently than any other gait type, there were significant differences among individuals in limb phase values (*F* (4,437) = 77.2, *p*<0.001) when tested across overlapping ranges of duty factor (0.65–0.80; see methods). Based on a Bonferroni multiple comparisons test, Participant 1 used significantly lower limb phases on average than all other participants (*p*<0.001), except Participant 5 (*p*>0.05). Participant 2 used significantly higher limb phases than all other participants (2 vs. 1,4 and 5, *p*≤0.001; 2 vs. 3, *p* = 0.002). Participants 3, 4, and 5 did not differ in limb phase values (*p*>0.05) (Figs. 4,5).

Across the sample (*r* = 0.534, *p*<0.01), and within Participant 1 (*r* = 0.632, *p*<0.001), Participant 3 (*r* = 0.628, *p*<0.001), and Participant 4 (*r* = 0.334, *p* = 0.003), limb phase and duty factor were positively correlated, although these two variables were not significantly correlated in participants 2 and 5 (*p*>0.05). Since duty factor and speed are inversely related [38], these results suggest that when moving at faster speeds, participants used lower limb phases, and shifted more toward lateral couplets, varying their gaits from LSDC to LSLC (Fig. 5).

### Additional cases

Of the five (symmetrical) strides we were able to quantify from available videos, two were categorized as LSDC, two as DSDC (one of these strides has a duty factor of 44% which indicates a running gait and is not as relevant to the analysis as are walking gaits), and one as a (walking) trot (Participants 6 and 7 in Fig. 5). Although this sample is very limited, it confirms that (as was shown for Participants 1–5), DSDC gait may occasionally be used, but is not the only gait preferred by human individuals walking quadrupedally.

## Discussion

### Lateral vs. diagonal sequence

Our quantitative gait analysis refutes the repeated claim by Tan and colleagues that individuals purported to have “Uner Tan Syndrome” (or who otherwise frequently or habitually use quadrupedalism) share the gait type characteristic of nonhuman primates. Although nonhuman primates exhibit flexibility in quadrupedal walking preferences based on developmental stage [4], [49]–[54] or substrate orientation [55]–[59], adult nonhuman primates walking on level surfaces highly prefer walking gaits that combine diagonal couplets (hind and forelimbs from opposite sides of the body swing and land close together in time) with diagonal sequence (forelimb lands after contralateral hind limb, and before ipsilateral hind limb) [4]–[8], [34]–[37]. In contrast, the human participants analyzed here nearly exclusively used lateral sequence walking gaits (forelimb lands after ipsilateral hind limb, and before contralateral hind limb; Video S1), combined with either diagonal or lateral couplets, with the most frequent gait being LSDC.

### Comparisons to quadrupedalism in healthy adults, infants and children walking on hands and feet

In accordance with our results, previous studies have consistently reported that healthy adults walking on hands and feet in an experimental setting use lateral, not diagonal sequence gaits [45]–[48]. Therefore the nearly exclusive preference for lateral sequence (i.e., nonprimate-like) quadrupedal walking exhibited by adult humans with UTS is by no means remarkable or unexpected. A comparison of our results to those of other studies also reveals that while the interlimb *sequencing* in adult humans is very predictably defined as lateral, limb *coupling* tends to vary among and within individuals. For example, Sparrow [45] reported that five adult males walking on hands and feet (as did all individuals analyzed here), used lateral sequence gaits, in singlefoot or lateral couplets (Based on our slightly modified ranges for gait category (following [7]), we would categorize the limb phase values for the five participants reported in Sparrow [45] (estimated from his Fig. 8) as lateral sequence, diagonal couplets (Participants SK, DW), lateral sequence singlefoot (Participant SD), and lateral sequence, lateral couplets (Participants BR, E)). Sparrow [45] also calculated limb phase for an adult female photographed by Muybridge in the late 19^th^ century [60], which also fell into the LSLC range. Similarly, Patrick et al. (page 608 [47]) reported that five adults walking on hands and feet showed “little limb pairing to pace-like coordinations.” More specifically, their data (estimated from their Fig. 6), show limb phases ranging between approximately 12 and 32%, corresponding to gaits that would be categorized here as lateral sequence, lateral couplets (12–24%), lateral sequence singlefoot (25%), and lateral sequence, diagonal couplets (26–32%). Another recent study [48] further confirmed that healthy human adults walking on hands and feet use lateral sequence gaits, although individuals varied in the use of diagonal or lateral couplets. Like adults, infants and children walking on hands and feet use lateral sequence gaits [4], [47]. Within these lateral sequence gaits, children use either lateral or diagonal couplets [4], and infants prefer diagonal couplets or trots [47], [61]. Contra Tan et al. [18], the paralytic child photographed by Muybridge in 1901 is using lateral, not diagonal sequence. In the image depicted in [18] the left hind limb is about to make contact. If this were diagonal sequence, the right forelimb would still be in swing phase, but instead, it is in middle support phase, indicating that it has landed *before* the contralateral hind limb as occurs in lateral sequence. That this is an example of lateral sequence gait can also be confirmed by examining the videos that have been made from Muybridge’s frame by frame images (e.g. http://www.youtube.com/watch?v=VirEtlOAb5o).

Our data show that when using lateral sequence gaits, individuals with UTS displayed both lateral and diagonal couplets, but all individuals highly preferred diagonal couplets, in contrast to previous studies in which lateral couplets appear to be used with equal or greater frequency than diagonal couplets [45], [47], [48]. However, for three of the individuals in our study, limb phase values were positively correlated with duty factor, and thus inversely correlated with speed. In other words, at higher speeds (lower duty factors), LSDC gaits transitioned into LSLC gaits, as is particularly evident in Participant 1 for whom we measured the greatest number of strides (Fig. 5). Although we were not able to measure stride length for our data, if individuals with UTS increased speed by increasing hind limb stride length [62], [63], this would result in increased potential interference between ipsilateral fore and hind limbs. In lateral couplet gaits, ipsilateral fore and hind limbs swing forward as a pair, preventing potential ipsilateral limb interference, and thus LSLC is the gait of choice for long limbed mammals, particularly when moving quickly [7], [38], [47], [53], [64], [65]. Because human hind limbs are elongated in association with bipedal adaptation, the potential for ipsilateral limb interference is increased for humans using hands and feet (compared to hands and knees) quadrupedalism, explaining the preference for lateral couplets (LSLC) [47] or (as in the case for individuals with UTS ) the shift from LSDC to LSLC at higher speeds. At slower speeds, where limb interference may be less problematic, LSDC might be preferred over LSLC because the former provides more stability via broader support triangles during three-limbed support, emphasis on contralateral two-limbed support, and minimization of ipsilateral two-limbed support [7], [38], [65].

Although couplet preferences in individuals with UTS appear to be speed related, at least in some individuals, the overall difference in couplet preference between individuals with UTS analyzed here (diagonal couplets) vs. adults described in the literature (lateral couplets; [45], [47], [48] might not be solely a function of speed but might also be a function of substrate. In the studies by Patrick et al. [47] and Maclellan et al. [48], adults walked on a treadmill, whereas UTS participants moved overground. Studies of quadrupedalism in various mammals (and bipedalism in humans) have shown that limb kinematics can differ on treadmills compared to overground walking [e.g., 66–72]. For example, in anticipation of sudden stops of the treadmill, cats maintain speed by increasing stride length, thereby maximizing relative limb support duration, and thus, stability. As a result of increased stride length, cats switch from lateral sequence, diagonal couplets to lateral sequence, lateral couplets or a pace in order to counteract ipsilateral limb interference brought about by increased stride length [67]. If the adult humans moving on treadmills quadrupedally experienced similar adjustments to stride length and support duration, it could explain their preference for lateral couplets, in comparison to the diagonal couplets preferred by individuals with UTS.

### Comparisons to quadrupedalism in healthy adults, infants and children walking on hands and knees

Infants using hands and knees crawling exhibit lateral sequence, diagonal couplet gaits or trots [4], [44], [47], [61], [73]. Adults using hands and knees crawling use nearly exclusively lateral sequence gaits, but show variability in couplets, with some showing a preference for diagonal couplets, others showing a preference for lateral couplets, and others showing a wide range of couplets with no preference [47]. As noted above, diagonal couplets provide more stability than lateral couplets [7], [65], and in nonhuman quadrupeds, are preferred in shorter limbed animals in which ipsilateral limb interference is not an issue [64], [65]. Because hind limbs are “shortened” relative to forelimbs during hands and knees compared to hands and feet quadrupedalism in humans, this could explain the general preference for diagonal couplets (or the flexibility to use either diagonal or lateral couplets) in the former. Regardless of the variation in the limb phase values (and couplets) used when walking on hands and knees vs. hands and feet, it is clear from the literature that diagonal *sequence* gait is not (or only rarely) used by infants, children, or adults on either hands and knees or hands and feet (including those with UTS).

### Couplets vs. sequence

Tan and colleagues appear to have misidentified the quadrupedal gait sequences of individuals with UTS as primate-like by confusing diagonal *sequence* with diagonal *couplets*. The participants more frequently used diagonal couplets than lateral couplets, but as noted above, the *sequence* associated with the couplets was almost exclusively lateral. Therefore, references to “diagonal sequence” or “diagonal gait” made by Tan and colleagues in reference to still images or videos should either be corrected to “diagonal couplets”, or (with respect to strides using lateral couplets) should be considered wholly misapplied. Either way, the humans at issue should not be described as displaying nonhuman primate-like quadrupedalism.

Confusion between couplets and sequence has also led to erroneous attempts to link primate quadrupedal gait to “ancient” tetrapod locomotion, with statements such as “the neural circuits responsible for the diagonal-sequence QL [quadrupedal locomotion] have been preserved for about 400 million years since the first emergence of QL in the fishlike tetrapods” (page 82 [32]); and see [13], [18], [31]. The references cited in support of these statements [74], [75] point to the origin of diagonal couplets, or trots, not the origin of diagonal *sequence.* Therefore, although it is possible that the use of diagonal *couplets* can be traced to early tetrapods [38], [74], this does not explain why mammals (including humans) vary with respect to using lateral or diagonal couplets. Rather, as discussed above, each type of couplet has biomechanical advantages, with lateral couplets serving to avoid limb interference, and diagonal couplets providing stability. The use of diagonal couplets in adult humans walking quadrupedally can thus be explained on the basis of biomechanical considerations, without the need to invoke evolutionary “atavism”.

## Conclusions

We have shown that the quadrupedalism used by individuals with UTS resembles that of healthy human adults asked to walk quadrupedally in an experimental setting, and neither group prefers the diagonal sequence/diagonal couplets gait characteristic of nonhuman primates. Rather, human adults prefer lateral sequence walking and vary in the type of couplets, which we suggest are dependent on speed, substrate, or biomechanical constraints related to ipsilateral limb interference or overall stability. Thus, as with nonhuman mammals, quadrupedal gait preferences in humans are best understood as a function of biomechanical constraints, rather than genetic mutations. We agree with researchers who have pointed out that the use of habitual quadrupedalism in individuals with UTS is an adaptation to instability of the trunk caused by cerebellar dysfunction, combined with environmental conditions such as insufficient medical care (e.g., [25]–[30]).

Further, we are unconvinced by the recent argument that individuals who use quadrupedalism in the absence of neural deficits or ataxia constitute supporting evidence for “the reemergence of the ancestral diagonal quadrupedal locomotion” (page 1 [31]). First, the purported case studies used to support this claim consist of (1) a 12-year-old boy, who could walk and run upright with no difficulty but “preferred running on all fours for fast locomotion, such as during playing with his father or hurrying to the WC on waking at night,” and (2) a 28-year-old man with a *paralyzed* left leg since infancy, who habitually used quadrupedal locomotion, refusing assistance from crutches or a wheelchair [31]. These two cases do not support the claim of re-emergence of “ancestral locomotion.” A nondisabled child’s preference for running on all fours in some situations such as play, or the choice of an adult with a paralyzed leg to walk quadrupedally, is not evidence of anything but the ability of humans to move in a variety of ways other than bipedalism when needed or desired. In fact, this type of “adaptive phenotypic plasticity,” not associated with genomic change [76], [77] has also occurred in the opposite locomotor direction in nonhuman mammals. Examples include accommodation to habitual movement on two hind limbs in a goat born without forelimbs [78], a baboon with paralyzed forelimbs [76], a dog born with forelimb deformities [77], or otherwise quadrupedal primates trained to walk bipedally from a young age [79]. Certainly, the existence of animals that learn to walk bipedally due to necessity (e.g., due to forelimb deformity) or training would not be interpreted as representing “forward” evolution toward humans. For the same reason, we disagree with the implication that humans who use quadrupedalism represent a “reversal” to a primitive locomotor state. Rather, humans, as well as nonhuman animals, exhibit locomotor plasticity in whatever direction is available and biomechanically advantageous.

We conclude that although the habitual use of quadrupedalism by adults with UTS is unusual, the *form* of this quadrupedalism resembles that of healthy adults and is thus not at all unexpected. As we have shown, quadrupedalism in healthy adults or those with a physical disability can be explained using biomechanical principles rather than evolutionary assumptions.

## Supporting Information

Table S1 **Comparison of gait type frequencies in Family “A” using methods of Cartmill et al. [7] and Hildebrand [37].**
(DOCX)

Dataset S1 **Kinematic variables for all participants/strides.**
(XLSX)

Video S1 **Sample video of Participant 1, walking in lateral sequence, diagonal couplets. (limb phase = 0.28, mean duty factor = 0.67).**
(MP4)
