# Supplementary material for: Human Quadrupeds, Primate Quadrupedalism, and Uner Tan Syndrome
Source: PLoS One. 2014 Jul 16;9(7):e101758. doi: 10.1371/journal.pone.0101758 (PMC4100729; doi:10.1371/journal.pone.0101758)
Supplement: Table S1 — Comparison of gait type frequencies in Family “A” using methods of Cartmill et al. [7] and Hildebrand [37]. (DOCX) [file pone.0101758.s001.docx]

|  | Cartmill definition | | |
| --- | --- | --- | --- |
| Gait type | Frequency | Percent | Limb phase range |
| LSLC | 52 | 10.1 | > 0 and < 0.25 |
| LSSF | 18 | 3.5 | 0.25 |
| LSDC | 436 | 85.0 | > 0.25 and < 0.50 |
| TROT | 2 | 0.4 | 0.50 |
| DSDC | 5 | 1.0 | > 0.50 and < 0.75 |
| **Total** | **513** | **100** |  |
| **Total LS** | **506** | **98.6** |  |
|  |  |  |  |
|  |  |  |  |
|  | Hildebrand definition | |  |
| Gait type | Frequency | Percent | Limb phase range |
| LSLC | 8 | 1.6 | >0.0625 and < 0.1875 |
| LSSF | 175 | 34.1 | > 0.1875 and < 0.3125 |
| LSDC | 291 | 56.7 | > 0.3125 and < 0.4375 |
| TROT | 38 | 7.4 | > 0.4375 and < 0.5625 |
| DSDC | 1 | 0.2 | > 0.5625 and < 0.6875 |
| **Total** | **513** | **100** |  |
| **Total LS** | **474** | **92.3** |  |

Table S1. Comparison of gait type frequencies in Family “A” using methods of Cartmill et al. [7] and Hildebrand [38].

LS=Lateral sequence, DS=Diagonal sequence, LC=Lateral couplets, DC=Diagonal couplets, SF=Singlefoot
